# Advanced Nanomaterials in Biomedical Applications (2nd Edition)

**DOI:** 10.3390/nano14201668

**Published:** 2024-10-17

**Authors:** Goran N. Kaluđerović, Nebojša Đ. Pantelić

**Affiliations:** 1 Department of Engineering and Natural Sciences, University of Applied Sciences Merseburg, Eberhard-Leibnitz-Str. 2, 06217 Merseburg, Germany; 2 Department of Chemistry and Biochemistry, Faculty of Agriculture, University of Serbia, 11080 Belgrade, Serbia

**Keywords:** nanomaterials, nanotechnology, biomedical application, drug delivery, therapeutics, diagnostics, imaging

## 1. Introduction 

Scientific research into new functional materials for nanotechnology has attracted worldwide interest, driving substantial efforts to discovering a variety of nanomaterials. These materials exhibit unique optical, magnetic, electrical, thermal, and catalytic properties, making them highly valuable in various fields, including pharmaceuticals and medicine. In particular, nanotechnology has emerged as a transformative force in medicine, particularly in drug delivery systems where nanoparticles can enhance the targeting and release of therapeutics, improving patient outcomes. Beyond cancer therapy, nanotechnology is being applied for targeted imaging, regenerative medicine, and the development of advanced diagnostic tools, allowing for earlier detection and treatment of diseases. Moreover, nanotechnology is playing a pivotal role in advanced imaging techniques, where nanoparticles can improve the contrast and resolution of imaging modalities such as MRI and PET scans. In regenerative medicine, nanomaterials are being explored for their ability to promote tissue engineering and wound healing. Additionally, innovative diagnostic tools utilizing nanosensors are enabling earlier detection of diseases, offering the potential for more effective interventions.

As the potential applications of nanomaterials continue to expand, the demand for the design and fabrication of novel nanoparticles is growing rapidly. This urgency is reflected in the Special Issue entitled “Advanced Nanomaterials in Biomedical Applications (2nd Edition)” which includes five articles, including two comprehensive reviews. These contributions delve into the latest advancements in nanomaterial synthesis and characterization, as well as their technological applications in various biomedical contexts.

In the following sections, we provide a brief overview of the key findings presented in this Special Issue, highlighting innovative approaches and breakthroughs that are paving the way for the next generation of biomedical applications using nanotechnology.

## 2. An Overview of Published Articles

The effectiveness of a drug is highly dependent on its administration method, which ideally should reduce enzymatic degradation, prevent harmful side effects, and improve bioavailability, whereas traditional delivery methods such as tablets or intravenous solutions can lead to issues such as overdose and inefficient pharmacokinetics. New drug delivery strategies utilize polymeric microsponges (PMS) to encapsulate drugs, enabling continuous release through slow diffusion or controlled degradation, while offering advantages such as safe release, prolonged administration, low-cost manufacturing, and improved thermal and physicochemical stability. In this context, Ariaudo et al. [1] prepared porous polysaccharide-based microsponges composed of cross-linked alginate polymers and characterized them by optical spectroscopy and nanoscopic microscopy techniques. The results have shown that macropores with a size distribution of 50 to 120 nm facilitated efficient loading and delivery of the therapeutic peptide CIGB814, which is currently in phase 3 clinical trials for the treatment of rheumatoid arthritis. The alginate microsponges exhibited 80% loading capacity and sustained peptide release through a diffusional mechanism, highlighting the potential of alginate’s edible and biocompatible nature for creating new carriers for controlled peptide drug delivery through alternative routes.

Madaci and co-authors [2] have developed a microconductometric sensor based on a chitosan composite containing the enzyme alcohol dehydrogenase (ADH) and its cofactor, the oxidized form of nicotinamide adenine dinucleotide (NAD^+^), as well as gold nanoparticles (GNPs). The sensor’s analytical performance was evaluated by measuring the headspace over aqueous solutions of ethanol and other solvents, such as methanol and acetone. The authors also used the ethanol sensor to detect ethanol in a commercial mouthwash and to detect ethanol vapor in the operator’s mouth after using a mouthwash and rinsing the mouth with water. This study indicated that the addition of gold nanoparticles is crucial in improving the analytical performance of the ethanol sensor in terms of response, sensitivity, selectivity, and reproducibility. The authors conclude that the ethanol sensor produced is of interest for personal use due to its simple application.

The dynamics of repeated vaporization of perfluorocarbon nanodroplets, sub-micrometer emulsions composed of a surfactant-encased perfluorocarbon, was described by Zhao et al. [3]. In this study, the nanodroplet shells were found to have an impact on the vaporization threshold as well on imaging half-life. The research results gained herein present a base for the further development of nanodroplets.

The review by Talukdar et al. [4] reports on a nanoparticle-based therapy for endometriosis, a painful and challenging condition for many women. The authors provide an overview of how such particles could improve treatments by offering more targeted and less aggressive solutions compared to current treatment options. From gene therapy to magnetic hyperthermia, these technologies have the potential to improve both diagnosis and treatment, providing hope for better outcomes and a higher quality of life for those suffering from this condition.

Lozovoy et al. [5] describe the advancements and applications of silicon-based avalanche photodiodes (APDs) in medical imaging. Such technology offers faster and more accurate detection of signals compared to traditional photodetectors, providing improved detection capabilities. The principles and features of APDs are also presented. This review highlights how APDs can improve critical imaging techniques such as positron emission tomography, single-photon emission computed tomography, time-of-flight positron emission tomography, computed tomography, fluorescence imaging and optical coherence tomography. With ongoing advances, especially in silicon-based APDs, these innovations could significantly enhance diagnosis and treatment of a variety of medical conditions, making healthcare more effective for patients.

## 3. Conclusions

The “Advanced Nanomaterials in Biomedical Applications (2nd Edition)” Special Issue highlights the significant progress and potential of nanotechnology in various medical fields. The contributions emphasize the unique properties of nanomaterials that enable advancements in drug delivery systems, targeted imaging, and innovative diagnostic tools. With ongoing research focused on the synthesis and characterization of novel nanoparticles, the potential for improved patient outcomes and early disease detection continues to expand. As these technologies evolve, they pave the way for the next generation of biomedical applications, promising to enhance therapeutic efficacy and transform healthcare practices.
